# Complex inguinal herniation containing a gravid uterus in a bitch – successful surgery and neonatal survival: a case report

**DOI:** 10.1186/s12917-026-05527-3

**Published:** 2026-05-01

**Authors:** Melchior Matyaszczyk, Natalia Sowińska, Marta Piątek, Szymon Kotwica

**Affiliations:** 1 MelKaVet Veterinary Clinic, Poznańska 15, Karpicko, 64-200 Poland; 2 Department of Internal Diseases and Diagnostics, Faculty of Veterinary Medicine and Animal Science, Poznan University of Life Sciences, Szydłowska 50, Poznań, 60-656 Poland; 3 Department of Animal Anatomy, Faculty of Veterinary Medicine and Animal Science, Poznan University of Life Sciences, Wojska Polskiego 71C, Poznań, 60-625 Poland

**Keywords:** Canine, Reproduction, Uterus, Pregnancy, Surgery, Herniorrhaphy, Ovariohysterectomy, Imaging

## Abstract

**Background:**

Inguinal herniation containing a gravid uterus, also referred to as inguinal gravid hysterocele (IGH), is an uncommon clinical condition in dogs characterized by protrusion of the pregnant uterus through the inguinal canal. Only a few cases have been documented, and most were diagnosed in early gestation or following complications such as fetal death. To our knowledge, reports of IGH diagnosed close to term and successfully managed surgically, resulting in live offspring, are limited.

**Case presentation:**

A 7-year-old, 16 kg, intact mixed-breed female dog presented with a large, non-painful inguinal mass. Diagnostic imaging revealed a left-sided inguinal hernia containing the uterus with two viable fetuses, intestinal loops, and the spleen. Ultrasonographic biometry suggested day 55 of gestation, consistent with advanced pregnancy. Physical examination and hematological and biochemical parameters were within reference ranges. Based on stable clinical findings and fetal viability assessment, a cesarean section combined with herniorrhaphy and simultaneous ovariohysterectomy was scheduled two days later. Both fetuses were delivered alive and initially showed good vitality. Postoperative recovery of the dam was uneventful, with no recurrence of herniation. One neonate died at 14 days postpartum, whereas the second survived and was successfully weaned.

**Conclusions:**

This case highlights the importance of thorough diagnostic evaluation and strategic surgical planning in managing IGH. This represents one of the few reported cases of IGH diagnosed at a late stage of gestation and managed at term without prior surgical correction, demonstrating that favorable maternal and neonatal outcomes are achievable with timely intervention.

**Supplementary Information:**

The online version contains supplementary material available at 10.1186/s12917-026-05527-3.

## Background

Hernias represent a significant clinical condition in veterinary medicine and are defined as the protrusion of an organ or tissue through an opening that may be natural or acquired [1, 2]. This pathological condition affects virtually all animal species and presents considerable diagnostic and therapeutic challenges for veterinary practitioners. The complexity of hernias lies not only in their varied presentations but also in their potential for serious complications, making early recognition and appropriate management crucial for optimal patient outcomes.

Hernias can be classified in several ways, depending on the time and location of their occurrence, as well as the circumstances under which they develop, which provides important information regarding their pathophysiology. One of the primary classification methods is based on their location. The most commonly recognized types of hernias include umbilical, inguinal, scrotal, femoral, perineal, and diaphragmatic hernias [2]. In small animals, inguinal hernias are considered a type of caudal abdominal hernia and are characterized by the protrusion of abdominal organs or tissues into the inguinal canal [1, 3]. From a temporal perspective, hernias are broadly categorized as either congenital or acquired. Hernias can also be classified as traumatic or non-traumatic based on their underlying cause. The distinction between these categories is important for understanding risk factors and implementing appropriate preventive measures. Inguinal hernias have been reported to occur more frequently on the left side, accounting for approximately 71.4% of cases, although the exact reason for this laterality remains unclear [3].

Sex is an important factor in the development of inguinal hernias. Acquired inguinal hernias are reported predominantly in females, whereas congenital hernias occur more often in males [3, 4]. The male predisposition to congenital hernias is thought to be related to delayed narrowing of the inguinal ring during testicular descent or to structural defects of the inguinal region that permit subcutaneous involvement of abdominal organs. In contrast, acquired hernias in females are associated with factors such as elevated estrogen levels, increased intra-abdominal pressure during pregnancy, trauma, or generalized tissue weakness [5]. Additional anatomical and metabolic influences also contribute: the inguinal canal in bitches is relatively wide, estrogen can further weaken the inguinal ring, and metabolic abnormalities may compromise abdominal wall integrity [6].

The diversity of organs and tissues that can become herniated reflects the complexity of this condition. The small intestine, colon, urinary bladder, spleen, and uterus are among the most commonly reported hernial contents [5, 7]. In female dogs, the uterus represents the most frequently herniated organ, with studies indicating that uterine herniation is reported in approximately 68% of cases compared with 41% for the small intestine and 5% for the colon [3]. The higher incidence of uterine herniation is attributed to its anatomical proximity to the inguinal canal via the round ligament of the uterus, which connects the uterine horn directly to the canal, combined with the shorter and wider inguinal canal in females, facilitating protrusion. Additionally, estrogen-mediated weakening of local connective tissues further predisposes the uterus to herniation compared with other organs such as the small intestine or colon. The herniation of reproductive organs is particularly significant, as it can lead to specialized conditions such as inguinal gravid hysterocele (IGH) when the gestational uterus protrudes through the hernial ring [7].

The clinical presentation of hernias, particularly inguinal hernias, can be confused with several other conditions, making accurate diagnosis challenging. Malignant mammary gland tumors, mastitis, inguinal lymph node hypertrophy, lipomas, hematomas, granulomas, and local abscesses are among the primary differential diagnoses that must be considered [3, 7, 8]. The similarity in clinical presentation between these conditions emphasizes the importance of thorough diagnostic evaluation, including advanced imaging techniques such as ultrasonography, to establish a definitive diagnosis.

The clinical significance of hernias extends beyond their immediate physical effects. These conditions can range from asymptomatic presentations to life-threatening emergencies, depending on factors such as the size of the hernial defect, the nature of the herniated contents, and the presence of complications such as incarceration or strangulation of the herniated organ [3]. The potential for serious complications, combined with the diagnostic challenges posed by their varied presentations, underscores the importance of systematic evaluation and appropriate management strategies. Understanding the classification, potential contents, and differential diagnoses of hernias provides the foundation for effective clinical management and optimal patient outcomes. This knowledge is particularly crucial when dealing with complex cases involving reproductive organs, where both maternal and fetal considerations must be balanced in treatment decisions.

### Case presentation

**Fig. 1:**
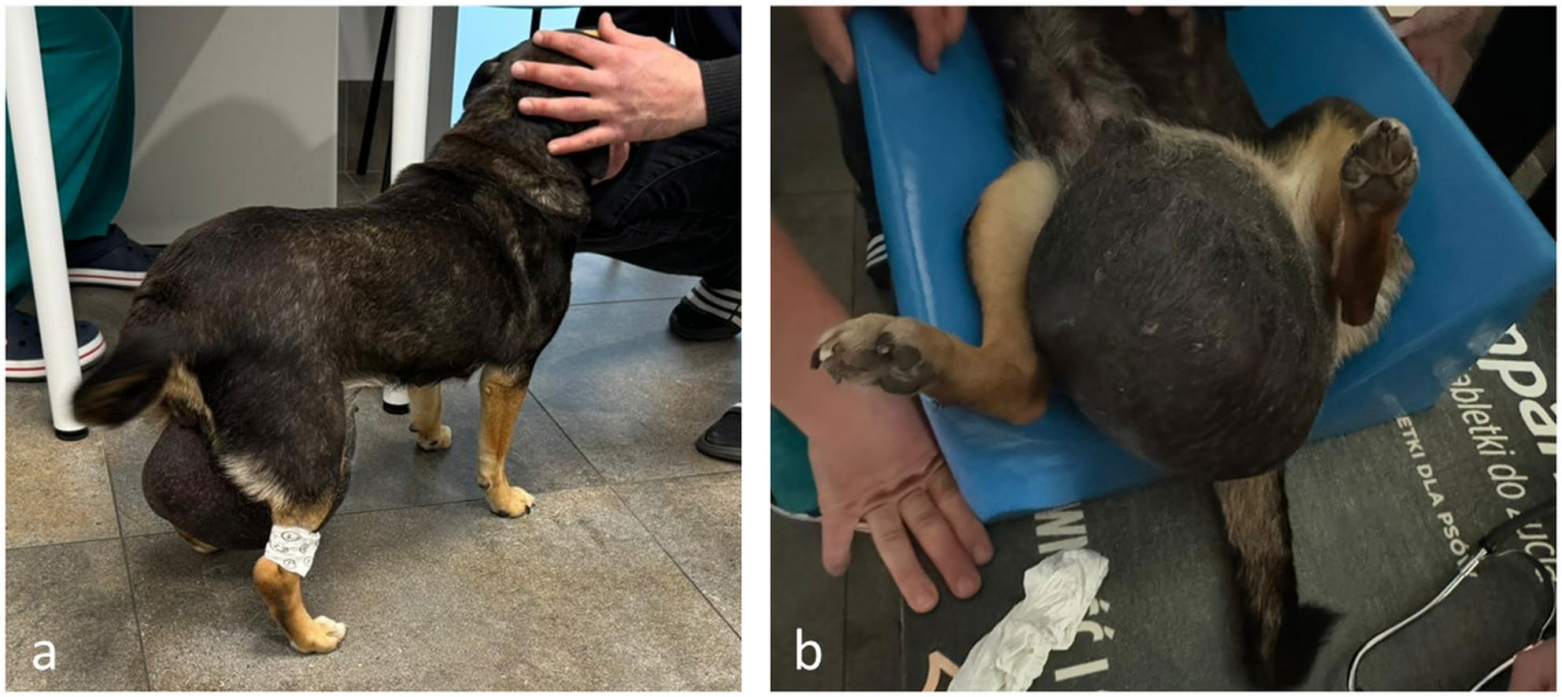
Photograph of the patient showing a large mass located between the hind limbs in a standing position (**a**) and in dorsal recumbency (**b**)

A 7-year-old, 16 kg, intact mixed-breed female dog of unknown reproductive history, phenotypically resembling a medium-sized short-haired shepherd-type dog, was found wandering on the street, subsequently adopted by the current owners, and later presented to the veterinary clinic for evaluation. The dog presented with a large, non-painful mass located in the caudal abdomen, initially suspected to be a neoplastic lesion. The mass measured approximately 20 cm in diameter, reached the ground, and occupied the space between the pelvic limbs (Fig. 1a and b). On clinical examination, the dog was bright, alert, showed no signs of pain, and exhibited normal physiological functions—appetite, urination, and defecation were unaffected. The patient’s neurological responses were normal, and the patient was calm and responsive during the physical and diagnostic assessments. Hematological and biochemical analyses revealed no clinically significant abnormalities. Complete blood count and serum biochemical parameters, including renal and hepatic markers, were within reference ranges (Table 1).

**Table 1:** Hematological and serum biochemical parameters of the bitch at presentation

Parameter	Result	Unit	Reference range
Albumin (ALB)	2.5	g/dL	2.2–3.9
Alkaline phosphatase (ALP)	136	U/L	23–212
Alanine aminotransferase (ALT)	60	U/L	10–125
Amylase	495	U/L	500–1500
Blood urea nitrogen (BUN)	19	mg/dL	7–27
Cholesterol	217	mg/dL	110–320
Creatinine	0.6	mg/dL	0.5–1.8
Gamma-glutamyl transferase (GGT)	0	U/L	0–11
Glucose	109	mg/dL	70–143
Lipase	941	U/L	200–1800
Phosphorus	3.8	mg/dL	2.5–6.8
Total bilirubin (TBIL)	< 0.1	mg/dL	0–0.9
Total protein (TP)	6.0	g/dL	5.0–8.0
Globulin (GLOB)	4.1	g/dL	2.5–4.5
Erythrocytes (RBC)	7.33	T/L	5.5–8.5
Hematocrit (HCT)	50	%	37–55
Hemoglobin (HGB)	17.9	g/dL	12.0–18.0
Leukocytes (WBC)	8.2	G/L	6.0–17.0
Platelets (PLT)	259	G/L	200–500

**Fig. 2:**
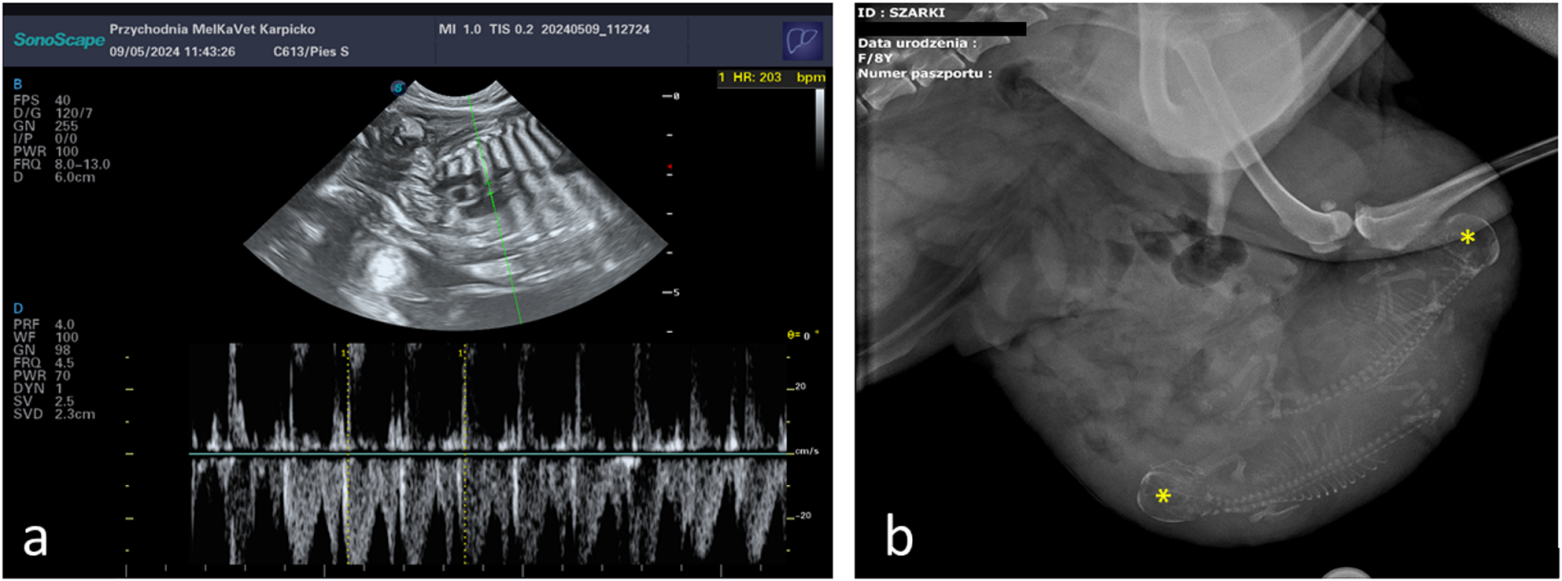
Ultrasonographic examination of the fetal heartbeat using pulsed-wave Doppler with a microconvex probe with the use of SonoScape P40 ultrasound system (**a**) and radiograph of the bitch showing the inguinal hernia region with visible skeletal structures of two fetuses (**b**). Fetal skulls are marked with asterisks (*). The radiograph was taken with the patient in right lateral recumbency

Based on anatomical localization and palpation, an inguinal hernia on the left side was suspected. Diagnostic imaging was performed to further evaluate the condition (Fig. 2a and b). Ultrasonographic examination of the inguinal mass confirmed that the hernial sac contained the uterus with two viable fetuses, small intestinal loops, and the spleen. Abdominal radiographs confirmed the presence of two fetuses, as evidenced by clearly visible fetal skeletons (Fig. 2b). Using a SonoScape P40 machine with a microconvex probe, an ultrasonographic examination was performed to conduct fetal assessment. Given the large size of the hernial mass and the advanced stage of pregnancy, and in the absence of pain or clinical signs of incarceration, manual reduction of the herniated contents was not attempted during clinical examination.

The detailed fetal assessment included biometric measurements, notably the biparietal diameter (BPD), which averaged 23.5 mm and correlated to approximately day 55 postovulation, on the basis of the formula of Luvoni and Grioni [9] for medium-sized breeds. Fetal heart rates measured with pulsed-wave Doppler ranged from 203 to 218 bpm in one fetus and from 232 to 250 bpm in the second fetus, indicating appropriate fetal vitality (Fig. 2a). Ultrasonographic evaluation revealed four-chambered fetal hearts, stomachs and urinary bladders, a distinct difference in echogenicity between fetal lungs and the livers, and intestines with weak but visible peristalsis. Although BPD suggests an early stage for elective cesarean section, the accuracy of this parameter decreases significantly in late gestation [10], with a possible error margin of ± 2 days. Considering these findings together with the stable clinical condition of the bitch, as evidenced by normal physical examination findings and hematological and biochemical parameters within reference ranges (Table 1), the timing of surgical intervention was determined based on a comprehensive obstetric assessment, including ultrasonographic evaluation of fetal viability, clinical examination of the dam, and the absence of signs indicative of imminent parturition or fetal distress. Surgical intervention was therefore scheduled for two days later.

**Fig. 3:**
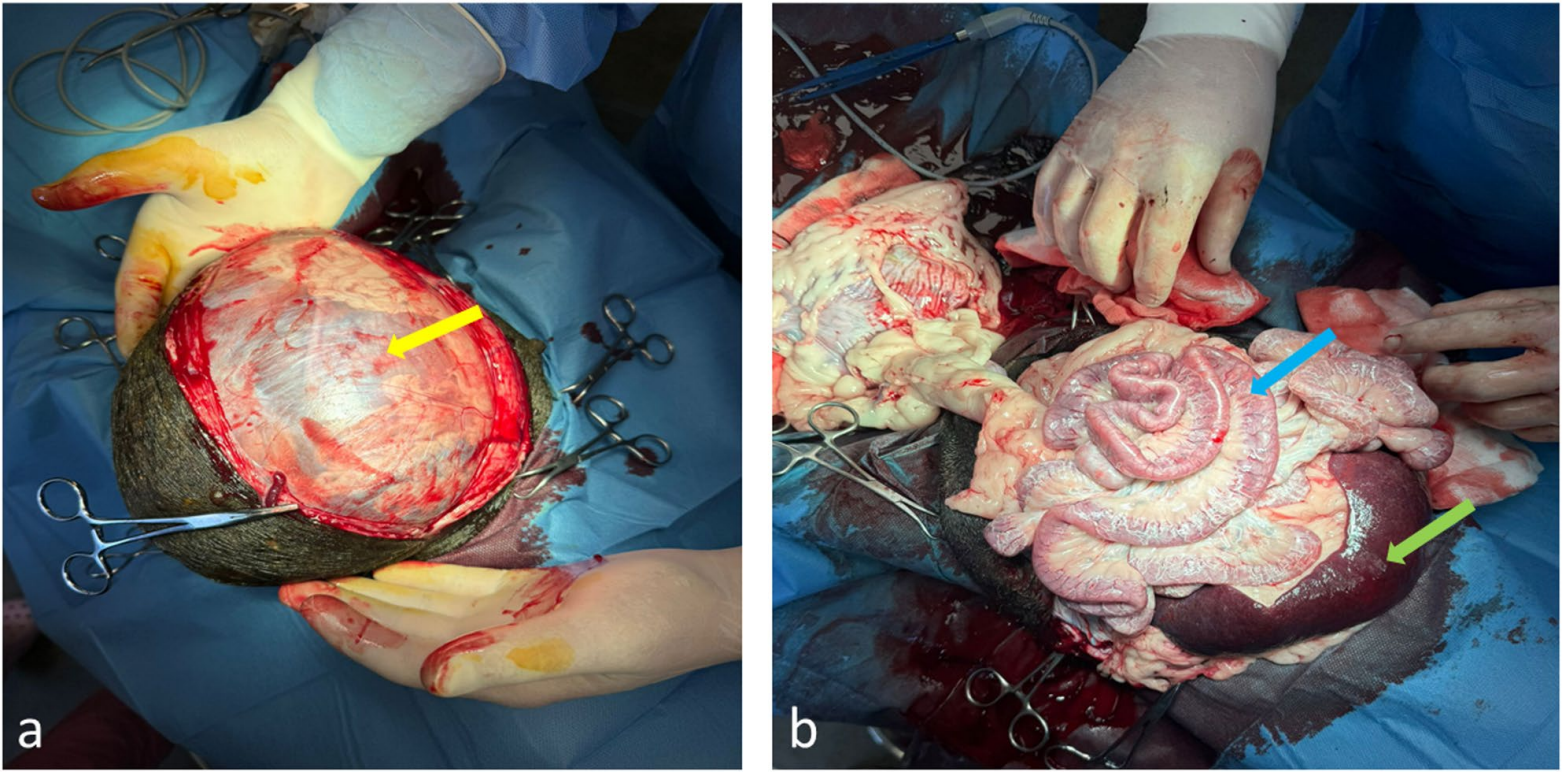
Intraoperative view of the inguinal hernia after skin incision, showing the uterine wall within the hernia sac (**a**, yellow arrow), and intraoperative view after removal of the gravid uterus, revealing intestinal loops (blue arrows) and the spleen (green arrow) within the hernia sac (**b**)

On the day of the scheduled surgery, the dog was placed under general anesthesia using dexmedetomidine (Dexdomitor^®^, Zoetis) at 0.001 mg/kg IV combined with methadone (Comfortan^®^, Dechra) at 0.2 mg/kg IV. Induction was achieved with propofol (Propomitor^®^, Dechra) at 2 mg/kg IV, and anesthesia was maintained with inhaled isoflurane delivered in oxygen via a standard anesthetic circuit, with an oxygen flow rate of approximately 1 L/min. Throughout the procedure, the patient received intravenous fluid therapy with Ringer’s lactate (Polpharma) administered at 5 ml/kg/h by continuous rate infusion. Postoperative analgesia included meloxicam (Melovem^®^, Dopharma) at 0.2 mg/kg SC on the first day, followed by 0.1 mg/kg PO once daily for the next three days. Under general anesthesia, the hernial sac was opened via the inguinal region (Fig. 3a). The hernial contents—namely, the uterus, small intestines, and spleen—were carefully repositioned into the abdominal cavity (Fig. 3b). A herniorrhaphy was performed to close the inguinal opening, and a simultaneous ovariohysterectomy was carried out to prevent future reproductive complications. A herniorrhaphy was performed to close the inguinal opening using a standard surgical technique as described by Fossum [1], with an absorbable monofilament suture (Monosyn^®^ 2/0; B. Braun, Melsungen, Germany). A simultaneous ovariohysterectomy was carried out to prevent future reproductive complications; the ovarian pedicles and the uterine stump were ligated using an absorbable monofilament suture (Monosyn^®^ 2/0; B. Braun, Melsungen, Germany). Subcutaneous tissues were closed with an absorbable monofilament suture (Monosyn^®^ 2/0; B. Braun, Melsungen, Germany) in a continuous pattern, and the skin was closed using surgical staples.

**Fig. 4:**
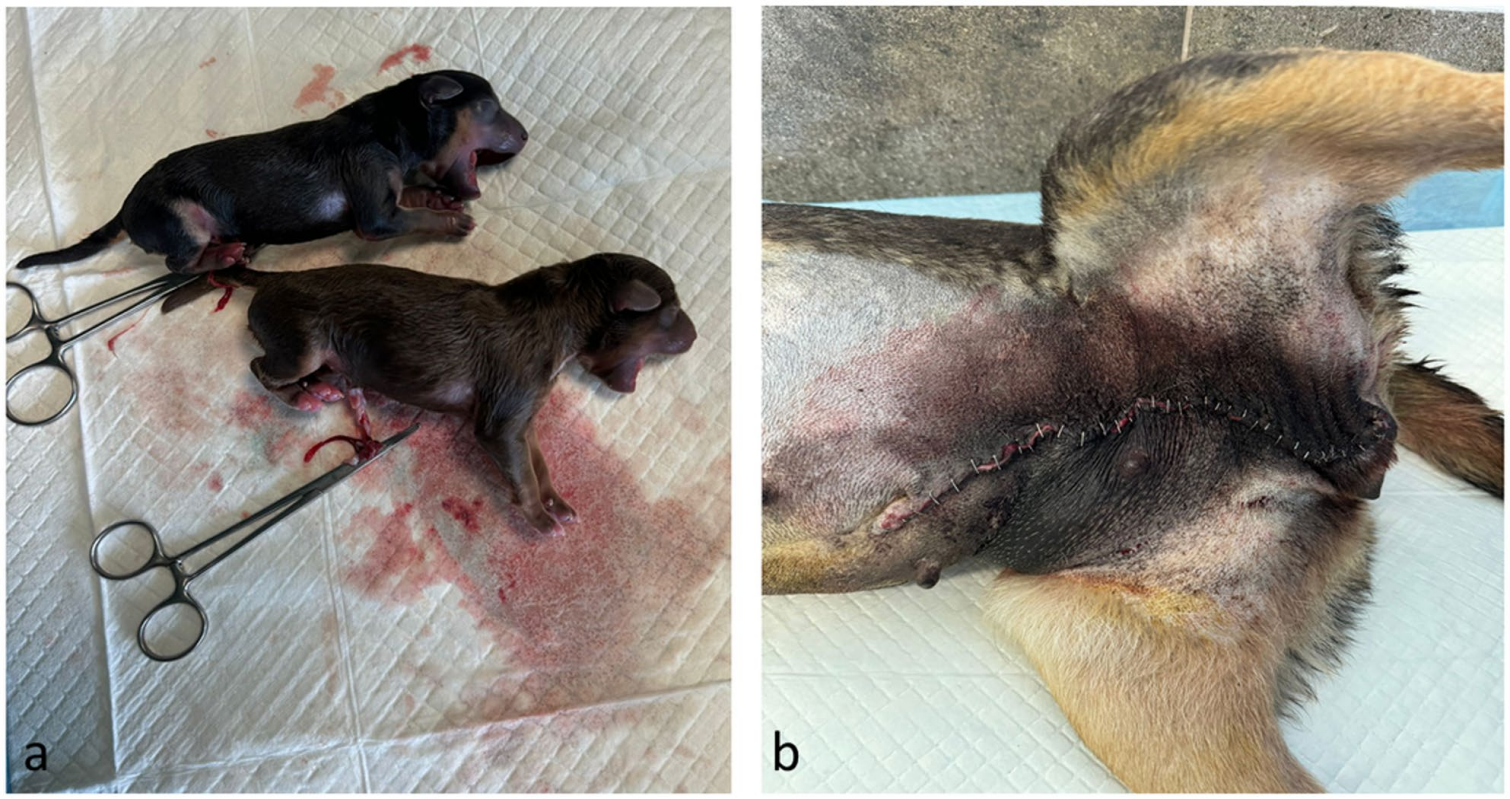
Photograph showing two neonates delivered by cesarean section performed within the hernia (**a**), and the postoperative wound closed with skin staples (**b**)

Both fetuses were delivered alive and required brief resuscitation (Fig. 4a). Open-mouth posture was visible immediately after delivery. Both neonates were viable at birth and required only brief standard neonatal supportive care, including drying, tactile stimulation, airway clearance, and thermal support. No pharmacological agents were administered. No additional preoperative or postoperative interventions specific to prematurity were considered necessary. The Apgar score was assessed according to the protocol described by Veronesi et al. [11] immediately after completion of initial neonatal resuscitation. Both newborns achieved an Apgar score of 10, indicating excellent vitality. Both neonates showed strong suckling reflexes and were placed with the dam for nursing, with colostrum intake observed shortly after birth. The birth weights of the neonates were 230 g and 245 g, respectively.

The postoperative outcome for the dam was uneventful, with good wound healing and no recurrence of herniation (Fig. 4b). Although the exact diameter of the hernial ring was not measured intraoperatively, it was subjectively assessed as markedly enlarged, allowing free repositioning of the herniated organs without signs of incarceration or strangulation. This observation may explain the progression of gestation despite the presence of a large inguinal hernia. Following surgery, the bitch and both neonates were monitored at the clinic for approximately 10 h. During this period, the neonates were provided with thermal support using an infrared heat lamp. They remained active, demonstrated effective suckling behavior, and maintained a body temperature within the expected neonatal range (approximately 35.5–37.0 °C). As the dam remained clinically stable and the neonates showed no abnormalities, they were discharged home. Continuous 24-hour hospitalization was not possible, as the clinic does not provide overnight care and the owners were unable to transfer the animals to an emergency facility. One neonate died at 14 days postpartum. Based on information provided by the owners, including the acute onset of respiratory signs and rapid clinical deterioration, cardiorespiratory failure was suspected; however, no post-mortem examination was performed, and the exact cause of death could not be definitively established. The second neonate survived and was successfully weaned at approximately 8 weeks of age. At the time of manuscript preparation (approximately 14 months postoperatively), both the dam and the surviving offspring were alive and reported to be in good health, with no observed long-term complications.

## Discussion and conclusions

Inguinal gravid hysterocele (IGH) is considered a rare condition in dogs, although several case reports have documented its occurrence in recent years [3, 5, 7, 8, 12, 13]. The majority of these cases were diagnosed either at midgestation or following complications such as uterine incarceration, fetal death, or the need for emergency intervention [5, 8]. In certain reports, advanced pregnancy in the presence of IGH has been associated with compromised uteroplacental perfusion and reduced fetal viability [5, 8].

Peinado et al. [7] described a case of inguinal gravid hysterocele managed surgically during mid-gestation, after which the pregnancy progressed and the puppies were delivered naturally. More recently, Veiga et al. [14] reported early surgical repair of IGH during early gestation, followed by elective cesarean section at term. In contrast, in the present case, the inguinal hernia was diagnosed at a late stage of gestation close to term. Surgical management was therefore performed at term and combined with cesarean section, resulting in the birth of live offspring. To the best of our knowledge, this case represents a rare clinical scenario of IGH maintained until late gestation and managed at term with combined hernia repair and cesarean section, resulting in the birth of live offspring.

Although previously reported cases of IGH have described adverse outcomes related to uterine entrapment and impaired fetal blood supply [5, 8], timely intervention in the present patient prevented such complications. The advanced stage of pregnancy facilitated clinical decision-making. The timing of the cesarean section was determined based on a complete obstetric assessment, including ultrasonographic evaluation of fetal heart rates and maturity, as well as clinical signs consistent with term pregnancy, such as mammary gland development and colostrum presence. These findings allowed the procedure to be scheduled two days after diagnosis without compromising fetal survival.

This case also underscores the importance of advanced imaging in differentiating IGH from other inguinal pathologies [3, 7]. Careful planning allows favorable maternal and neonatal outcomes despite the complexity of the condition. Although one neonate did not survive beyond the second week postpartum, the survival and successful weaning of the other puppy represent a favorable outcome compared with previously reported cases, in which fetal death or resorption associated with uterine herniation was described [5, 8, 12, 13].

In conclusion, this case contributes to the limited body of literature on IGH by documenting, for the first time, the successful management of a pregnancy diagnosed close to term. These findings emphasize the importance of accurate diagnosis, careful surgical planning, and appropriate perioperative care in achieving favorable outcomes.

## Supplementary Information

Supplementary Material 1.

## Data Availability

All the data generated during this study are included in this published article.

## Abbreviations

IGH: Inguinal gravid hysterocele
